# Cyberinfrastructure for Open Science at the Montreal Neurological Institute

**DOI:** 10.3389/fninf.2016.00053

**Published:** 2017-01-06

**Authors:** Samir Das, Tristan Glatard, Christine Rogers, John Saigle, Santiago Paiva, Leigh MacIntyre, Mouna Safi-Harab, Marc-Etienne Rousseau, Jordan Stirling, Najmeh Khalili-Mahani, David MacFarlane, Penelope Kostopoulos, Pierre Rioux, Cecile Madjar, Xavier Lecours-Boucher, Sandeep Vanamala, Reza Adalat, Zia Mohaddes, Vladimir S. Fonov, Sylvain Milot, Ilana Leppert, Clotilde Degroot, Thomas M. Durcan, Tara Campbell, Jeremy Moreau, Alain Dagher, D. Louis Collins, Jason Karamchandani, Amit Bar-Or, Edward A. Fon, Rick Hoge, Sylvain Baillet, Guy Rouleau, Alan C. Evans

**Affiliations:** ^1^McGill Centre for Integrative Neuroscience, Montreal Neurological InstituteMontreal, QC, Canada; ^2^Montreal Neurological InstituteMontreal, QC, Canada; ^3^Department of Computer Science and Software Engineering, Concordia UniversityMontreal, QC, Canada; ^4^McConnell Brain Imaging Centre, Montreal Neurological InstituteMontreal, QC, Canada; ^5^Douglas Mental Health University HospitalMontreal, QC, Canada

**Keywords:** neuroimaging, big data, open science framework, cyberinfrastructure, neuroscience, data sharing, bids, workflow

## Abstract

Data sharing is becoming more of a requirement as technologies mature and as global research and communications diversify. As a result, researchers are looking for practical solutions, not only to enhance scientific collaborations, but also to acquire larger amounts of data, and to access specialized datasets. In many cases, the realities of data acquisition present a significant burden, therefore gaining access to public datasets allows for more robust analyses and broadly enriched data exploration. To answer this demand, the Montreal Neurological Institute has announced its commitment to Open Science, harnessing the power of making both clinical and research data available to the world (Owens, 2016a,b). As such, the LORIS and CBRAIN (Das et al., 2016) platforms have been tasked with the technical challenges specific to the institutional-level implementation of open data sharing, including:

1. Comprehensive linking of multimodal data (phenotypic, clinical, neuroimaging, biobanking, and genomics, etc.)
2. Secure database encryption, specifically designed for institutional and multi-project data sharing, ensuring subject confidentiality (using multi-tiered identifiers).
3. Querying capabilities with multiple levels of single study and institutional permissions, allowing public data sharing for all consented and de-identified subject data.
4. Configurable pipelines and flags to facilitate acquisition and analysis, as well as access to High Performance Computing clusters for rapid data processing and sharing of software tools.
5. Robust Workflows and Quality Control mechanisms ensuring transparency and consistency in best practices.
6. Long term storage (and web access) of data, reducing loss of institutional data assets.
7. Enhanced web-based visualization of imaging, genomic, and phenotypic data, allowing for real-time viewing and manipulation of data from anywhere in the world.
8. Numerous modules for data filtering, summary statistics, and personalized and configurable dashboards.

Implementing the vision of Open Science at the Montreal Neurological Institute will be a concerted undertaking that seeks to facilitate data sharing for the global research community. Our goal is to utilize the years of experience in multi-site collaborative research infrastructure to implement the technical requirements to achieve this level of public data sharing in a practical yet robust manner, in support of accelerating scientific discovery.

## Introduction

The challenge of reproducibility in science (Campbell, 2016) has compelled the neuroscience research community to adopt new approaches to ensure scientific reliability without impeding innovation. The recent commitment by the Montreal Neurological Institute (MNI) to Open Science aims to improve replicability and transparency in research through collaboration, and in doing so, accelerate scientific discovery (Owens, 2016a,b).

The MNI's Open Science initiative calls for the free release of research data, findings, analytical tools, and publications from MNI-based researchers. Institutional sharing aims to prevent data loss, increase sample size and statistical power, and reduce acquisition costs by encouraging data re-use (thereby maximizing returns on public funding). In addition to these advantages, inviting external researchers to access these institutional resources will expand the reach and impact of research conducted at the institute (Poldrack and Gorgolewski, 2014).

Open Science initiatives have been spearheaded within the bioinformatics and neuroscience communities by groups such as the Center for Open Science (Asante et al., 2016), the Allen Institute (Koch and Jones, 2016), the Human Connectome Project (Van Essen et al., 2012), OpenfMRI (Poldrack et al., 2013), the Consortium for Reliability and Reproducibility (CoRR) (Zuo et al., 2014), and a multitude of independent data sharing and open-source academic software initiatives such as BrainHack (Craddock et al., 2016), Brainstorm (Baillet et al., 2011), SPM (Friston et al., 1994), FSL (Jenkinson et al., 2012), ADNI (Petersen et al., 2010), Nipype (Gorgolewski et al., 2011), and BigBrain (Amunts et al., 2013). At the same time, emerging definitions of common data sharing standards, practices, and formats are being established via BIDS (Gorgolewski et al., 2016), the Neuro-Imaging Data Model (NIDM) (Maumet et al., 2016), FAIR principles (Wilkinson et al., 2016) and even extending to data organization and citation strategies (Honor et al., 2016). Meanwhile, governments and funding agencies in the USA (National Institutes of Health, 2014; National Institute of Mental Health, 2015), Canada (Tri-Agency Statement of Principles of Digital Data Management, 2016), Europe (Horizon 2020, The Wellcome Trust, 2016) and elsewhere encourage and increasingly require research programs to establish data management and sharing plans from the start of the research data lifecycle. Despite these efforts, such initiatives are frequently constrained to particular projects or focused collaborations rather than institutional initiatives, as the sharing of data often remains at the discretion of individual investigators whose technical resources and expertise in data infrastructure may be limited.

As the first leading academic research institution to develop an Open Science framework at the institutional level^1^, the MNI's cyberinfrastructure platform will play a critical role in this initiative. To fulfill this vision, several key implementational challenges must be met, including policy, security, and ethics, as well as infrastructural design, software interoperability, data harmonization, validation, processing, and provenance capture. The solutions to these issues must adhere to open data sharing principles and respect domain-specific best practices (Honor et al., 2016; Nichols et al., 2016; Wilkinson et al., 2016).

For effective data sharing at an institutional level, it is imperative to use a cyberinfrastructure that can incorporate heterogeneous datasets acquired from multiple sources over time as well as across modalities – and to do so in a way that is robust. Data collected by investigators in multiple studies across the institute span diverse data types from many domains, including clinical/behavioral measures, biological samples from the MNI biobanking collections, genomic data, and a growing multimodal repository of brain imaging data. The institutional cyberinfrastructure housing these datasets must also be able to integrate workflows from all stages of the research data lifecycle, and interoperate with platforms that capture and disseminate large datasets.

To this end, the MNI has selected LORIS (Das et al., 2011) to serve as the core data management platform for this initiative, coupled to the CBRAIN distributed high-performance computing environment (Sherif et al., 2014). These two platforms, combined with embedded data visualization utilities (Sherif et al., 2015), constitute an “ecosystem” capable of supporting Open Science at an institutional level (Das et al., 2016).

This paper describes the ethical and policy challenges, the technical infrastructure used for storage and curation of the various data types, and the workflows and processing environment for the implementation of Open Science at the MNI.

## Methods

Four cornerstones of the MNI's Open Science framework and cyberinfrastructure are discussed below: (1) ethics (including subject privacy, consent and security), (2) multi-modal data entry, (3) workflows and quality control, and (4) high-performance data processing and software-systems interoperability.

### Ethics, privacy and security

Embarking on the endeavor of institutional Open Science poses unique challenges, particularly with regard to respecting ethical guidelines. One critical component is that personally identifiable information (PII) of all subjects must be protected and the data itself must be de-identified and secured within the context of private and independent databases—but will also be reconcilable into a single subject record in the Open Science platform.

Since the creation of the first human cell-line (Lucey et al., 2009), the ethical considerations surrounding the distribution and use of human subject data have been manifold (Nelson, 2015). In accordance with local Quebec law and research ethics, informed consent must be obtained from subjects in order to collect and study tissue and data. The Canadian Tri-Council has also provided clear criteria to protect the privacy of subjects, and these criteria must be met in order for researchers to have access to sensitive data (Canadian Institutes of Health Research Natural Sciences and Engineering Research Council of Canada and Social Sciences and Humanities Research Council of Canada, 2014). Accordingly, a proposal was submitted and approved by the MNI Research Ethics Board (REB) for the Neuro OpenScience Clinical Biologic Imaging and Genetic Repository, or C-BIG-R, addressing the implementation of an infrastructure technically compatible with these ethics policies. A dual-level governance structure was created to oversee these ethical concerns via the REB as well as a newly-established “Tissue and Data” committee. The REB is tasked with the identification of best practices employed by comparable initiatives, and the Tissue and Data committee is responsible for determining what materials are deposited into the bank, the storage mechanisms, and how they can be accessed for research. Participating studies may profit from this governance model throughout the research data lifecycle, since matters of storage, security, inclusion, and exclusion criteria, disposal of samples etc., will already be covered by this ethical framework.

**Figure 1:**
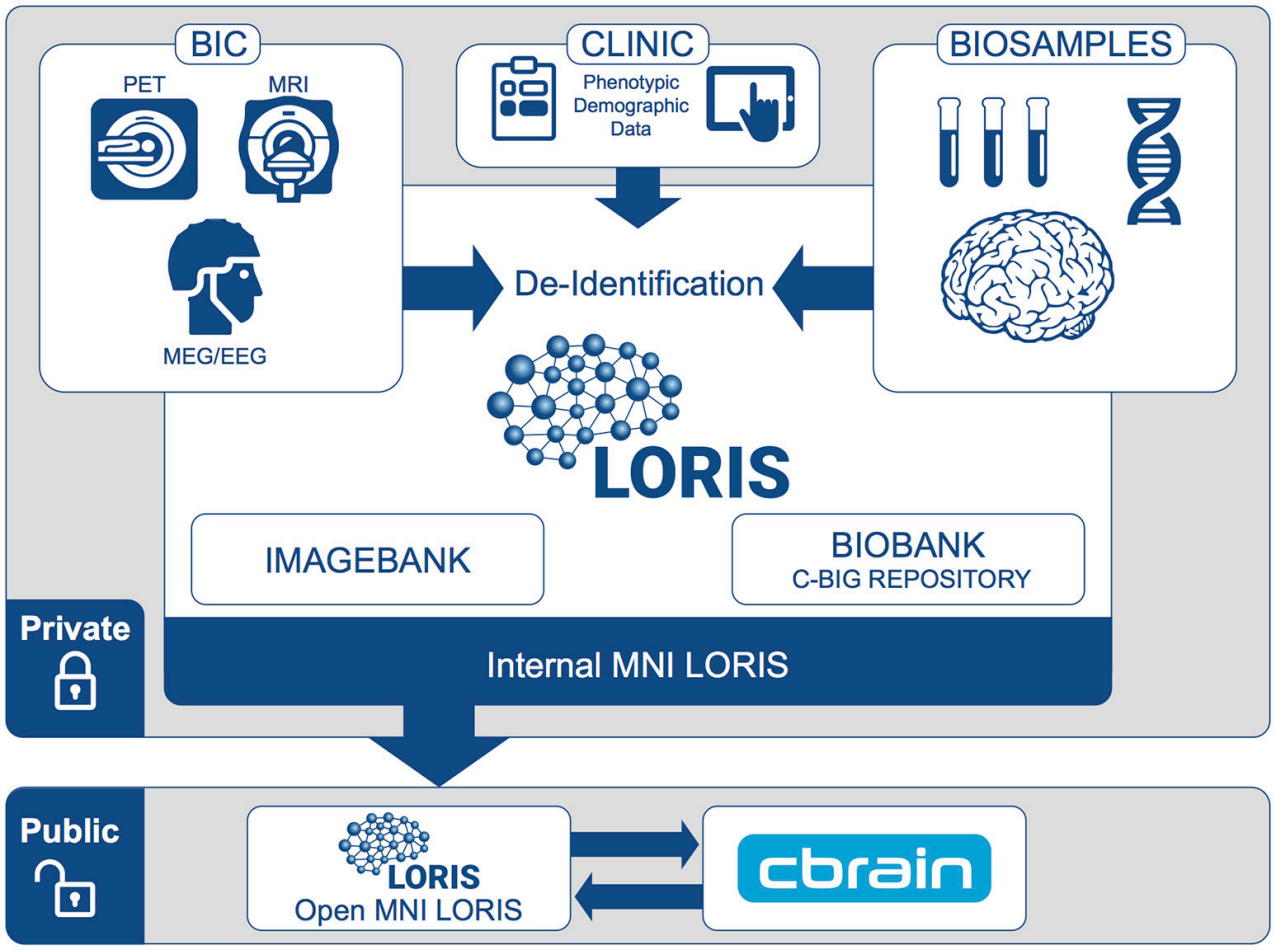
**MNI data flow from internal institutional repository to public-facing Open Science platform**. At the institutional level, data are organized within individual studies and are only accessible by users approved by the study's principal investigator. Subjects participating in multiple studies are assigned unique IDs for each study. When data are shared to the Open MNI repository, a subject's data will be linked across all studies by a new unique subject ID.

Data sharing at any level requires nuanced procedures and consent processes, and involves particular technological constraints. These technical considerations include how to share data (i) within a single study as well as (ii) between collaborating investigators, and finally (iii) at an institutional and public level such that subject data from multiple studies are linkable and queryable in a unified manner. From its inception, the MNI's platform design allows researchers to first store and share data internally and privately, while ultimately allowing data to be selectively pushed to the public-facing platform for dissemination (Figure 1). Both de-identification and reconciliation of subject records must be carefully designed in view of the Open MNI platform.

**Figure 2:**
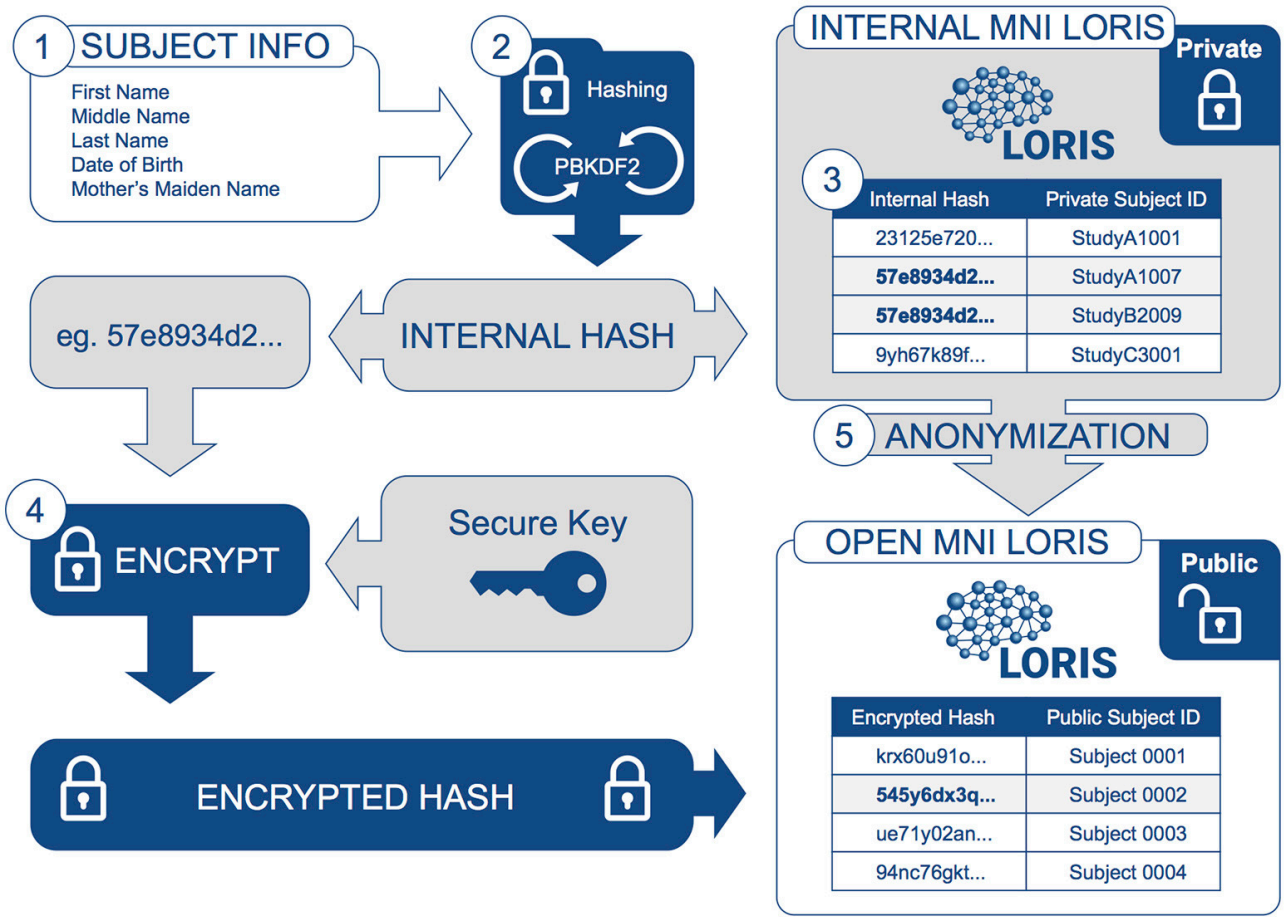
**Information Flow for De-identification: Identifying subject information is encrypted and protected at each step**. Subject information is collected (1) and then iteratively hashed (2) by a PBKDF2 algorithm using a SHA1 function to generate an Internal Hash value. This Internal Hash is mapped to a unique subject ID for each study (3); this mapping is stored in a database only accessible by database administrators (Internal MNI LORIS). Users of the Internal MNI LORIS platform will reference each study participant by this unique private ID, such that an individual enrolled in different studies will be registered under different subject IDs. For datasets that are selected for sharing via the Open MNI LORIS platform, (4) the Internal Hash value for each subject is encrypted again using a secure key known only to database administrators, such that data cannot be easily linked back to private subject IDs. At the same time, (5) data are further anonymized and images de-faced (facial features removed) during transfer from the Internal MNI platform to the public-facing Open MNI LORIS data platform.

De-identification of subject data is an integral requirement: the identifier must ensure privacy and ethically-compliant data sharing, while also preventing data duplication. For this purpose, a system of hashed identifiers has been designed to safeguard subject identity at every stage and prevent reconstructive subject identification. This process encodes identifying information and is incorporated into LORIS such that PII is never transmitted over a network; only the encoded information is used (Figure 2).

A one-way cryptographic hash function is employed to uniquely refer to individual subjects without revealing any of their identifying information. A given subject's first, middle and last names, date of birth and mother's maiden name are concatenated and passed through the PBKDF2^2^ (“Password-Based Key Derivation Function 2”) algorithm to generate a unique hash value, created by iteratively applying a SHA1^3^ (Secure Hash Algorithm 1) hashing function one million times. The resulting hashed value (a 125-character string) is then mapped onto a unique MNI-internal identifier (e.g., “StudyA1007”), distinctly generated for every study in which the subject is a participant. These study-specific identifiers can be disseminated without compromising the subject's privacy. The internal hash is only accessible by database administrators and is therefore also kept secret within the institution.

Research platforms or researchers that have access to a subject's private information will never store PII directly in the database; rather, they will automatically trigger this hashing function when registering subject data in LORIS. The function was selected for its efficiency given a sufficiently short execution time to perform mass registration of data, yet long enough such that brute-force attackers cannot identify subjects by repeated attempts to guess subject names. The entire process of hashing takes approximately 7 seconds on a current CPU.

Datasets can be shared (at the owner's discretion) by uploading to the public-facing Open MNI repository. The sharing process entails additional data curation steps for further de-identification, such as transforming images via de-facing to avoid identification based on facial features (Bischoff-Grethe et al., 2007). Another of these transformations is an encryption performed on the locally hashed identifiers. This encrypted hash is used to detect non-unique subjects for the sole purpose of avoiding redundancy (i.e., same subject appearing in different datasets). When a subject is determined to be unique within the Open Science repository, they are assigned a unique public ID which unifies their de-identified data from disparate studies.

In the event that a subject revokes consent, a database administrator has the capability of removing that subject from the Open MNI LORIS database using the unique public subject ID. Upon revocation, the physical data as well as the computer records will be destroyed and deleted. However, any derived datasets or results obtained through the analysis of biospecimens and data for which consent has been withdrawn will not be destroyed. This process complies with NIH-NDA standards and methodology regarding Global Unique Identifiers (Johnson et al., 2010), and is explicitly outlined in the biobank consent form.

### Loris functionality: multi-modal data entry, provenance, storage, and linking

The LORIS system (Das et al., 2011, 2016) was designed specifically for heterogeneous data acquisition, curation and dissemination. It is a web-based PHP/MySQL database, freely available on GitHub^4^ as open-source software. Its modular organization and support for multiple data modalities (including behavioral/clinical, neuroimaging, and genetic summary data) provide a flexible and robust platform for many types of multi-site studies and projects.

Within LORIS, data are organized based on subject profiles and longitudinal data-collection timepoints within a given study. After creating a de-identified profile of a subject, multiple modalities of data are associated to that subject and their corresponding timepoints. For example, data collected at a particular subject timepoint may include the acquisition of MRI and PET volumes, a collection of biospecimens, and a variety of other clinical measures. All of this information is associated to the subject within LORIS and can be easily retrieved, reviewed, and exported.

Data can be imported into LORIS from external software systems, such as laboratory information management systems (LIMS) that handle sample registration, tracking, and storage. Such systems export data in various formats, demonstrate different data transfer capabilities, and implement varying configurations in their Application Programming Interfaces (APIs). To ensure interoperability across this diverse range of systems, a series of processing scripts have been created in order to bridge the gap between LORIS and the heterogeneous outputs of these platforms.

Importation of data is best illustrated through examples from two contexts: imaging volumes and biospecimen information. The transfer, insertion and processing of imaging data is performed via a sequence of open-source scripts^5^ native to the LORIS platform. These scripts form a software “pipeline” that is installed on the server to automate the pre-processing and insertion of imaging datasets. In addition, a web-based imaging uploader integrated with these server-side scripts handles image uploading, filename anonymization validation, and interactive flagging of protocol verification checks. Once loaded in the database, imaging volumes become searchable and sortable in the Imaging Browser module. 3D visualization of volumes and morphological surfaces is natively embedded in the interface via the BrainBrowser^6^ tool used for quality control review of images (Sherif et al., 2015).

Another approach is presently being explored for LORIS to directly import multimodal data organized according to the emerging BIDS convention (Gorgolewski et al., 2016): data volumes would be pushed automatically from their respective acquisition sources (MRI scanners, PET cameras, MEG, and EEG arrays) into a central BIDS-compliant file system. This consists of structured folders containing raw and metadata information in simple JSON files. The new data entries would then be systematically imported and registered into the database after being detected by an automated daemon process that monitors further updates to the BIDS system.

For biospecimen data, a similar automated workflow has been implemented. Biosamples are collected and processed in a lab, at which time information about the sample collected (e.g., sample type, date of collection, etc.) and its current status (e.g., stage of processing, storage location) are registered within a third-party LIMS data system. Custom scripts are used to extract data based on archives of these data systems, simultaneously converting and normalizing the data for use within LORIS.

Once data are acquired and loaded in LORIS (through either manual data entry or automated pipeline scripts), researchers will be able to review and curate information using quality control tools and procedures assuring quality inputs to their analysis pipelines. Following data acquisition, review and curation, researchers can download, query, and disseminate datasets via LORIS' Data Querying Tool (DQT) which is built on a NoSQL framework (Katz et al., 2005) to enable fast and precise extraction of large datasets. Via the DQT, users can construct complex queries and apply custom filters in order to target populations and subsets of interest.

Common data description vocabularies are required to properly address the challenges of Open Science at a large scale. However, implementing a common vocabulary covering the range of concepts involved in studies conducted across the MNI will be a significant undertaking, and will be driven by the MNI's researchers as they seek to share their data in a common Open Science framework; convergence upon a usable solution will be challenging. LORIS is committed to the standardization of ontologies, and currently adopts a practical approach where (1) all the (DICOM) fields related to imaging data are preserved and made queryable, and (2) terms used for behavioral variables and biobanking studies are defined on a study-by-study basis, while their re-utilization is also promoted across studies, compliant (where possible) with conventions such as BIDS (Gorgolewski et al., 2016) or NDAR (Hall et al., 2012). Prospectively, LORIS plans to adopt ontologies under development by the NIDM initiative to formally and uniformly describe raw data, terms, workflows and derived data (Maumet et al., 2016), as well as open data citation standards such as those developed for neuroimaging (Honor et al., 2016). Further integration of domain-specific standards, such as MIABIS 2.0 developed for biobanking data by the BBMRI-ERIC network (Merino-Martinez et al., 2016), is a priority for integration of data dissemination formats for the Open Science platform.

### Workflows and quality control for imaging, clinical/behavioral and biobanking

**Figure 3:**
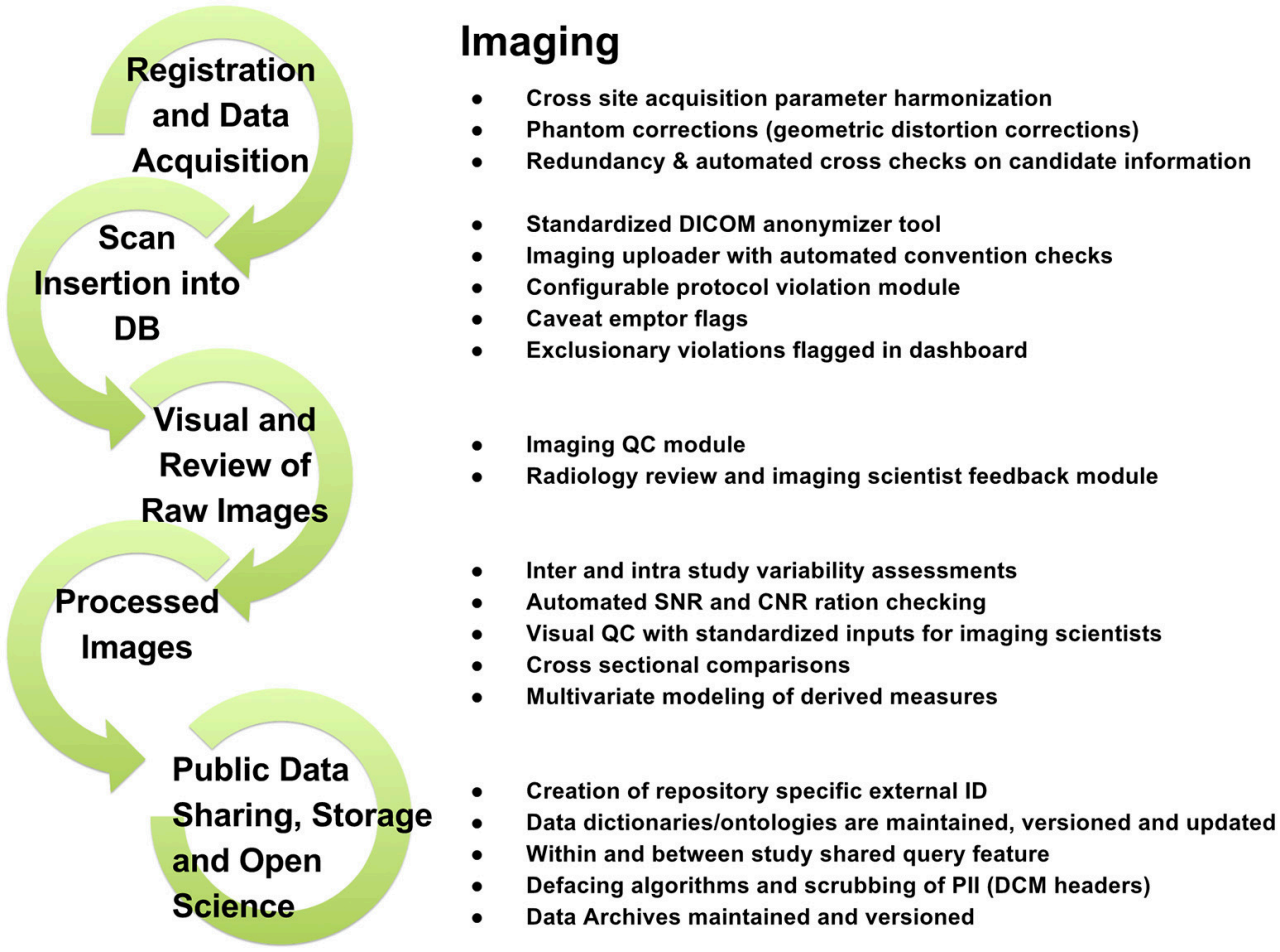
**Imaging workflow from subject registration to data sharing in Open Science detailing processes for radiological reviews, quality control, and dissemination**.

**Figure 4:**
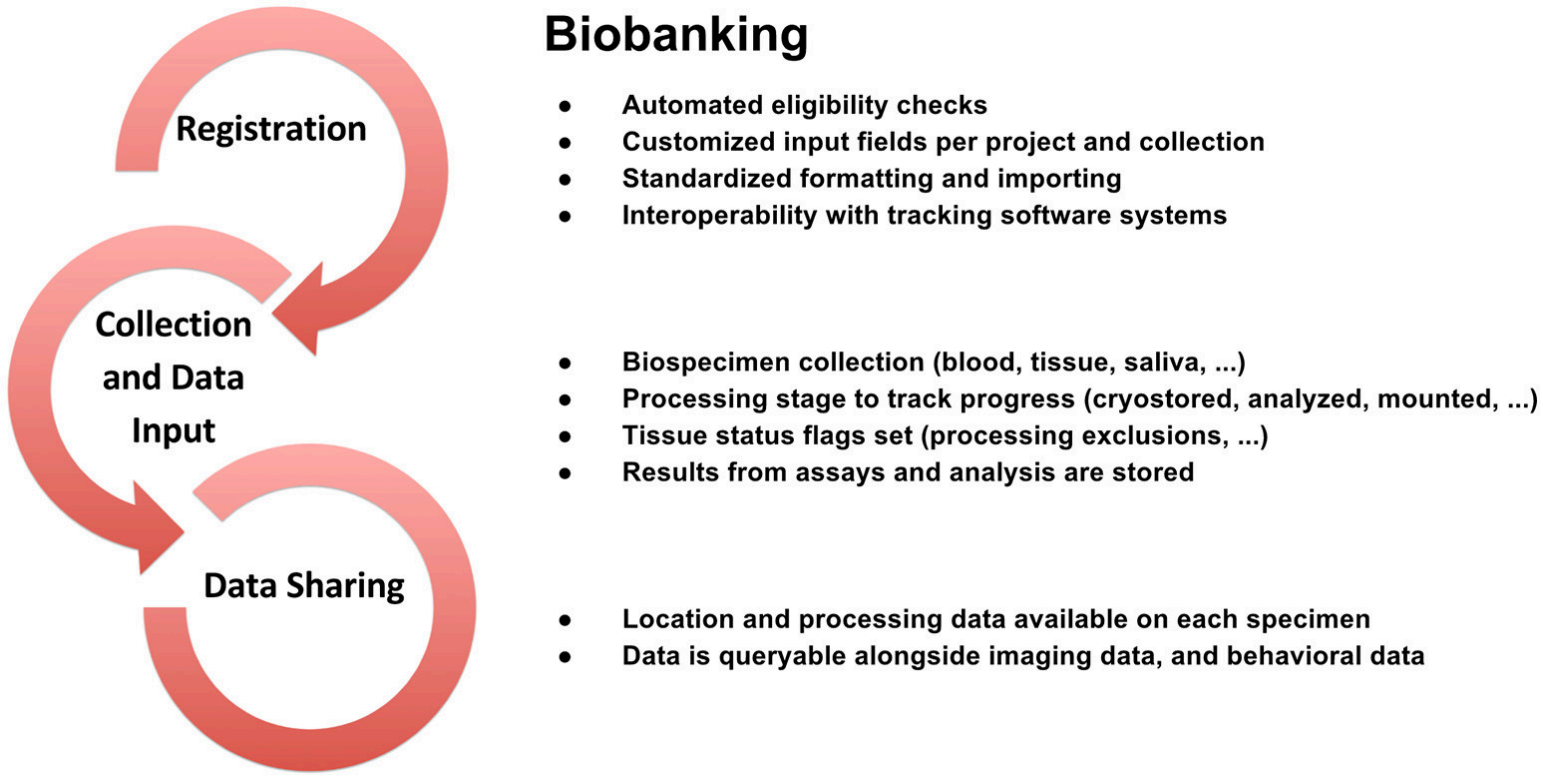
**Biobanking workflow from subject registration to data sharing in Open Science detailing data collection, sample tracking, quality control, and dissemination**.

**Figure 5:**
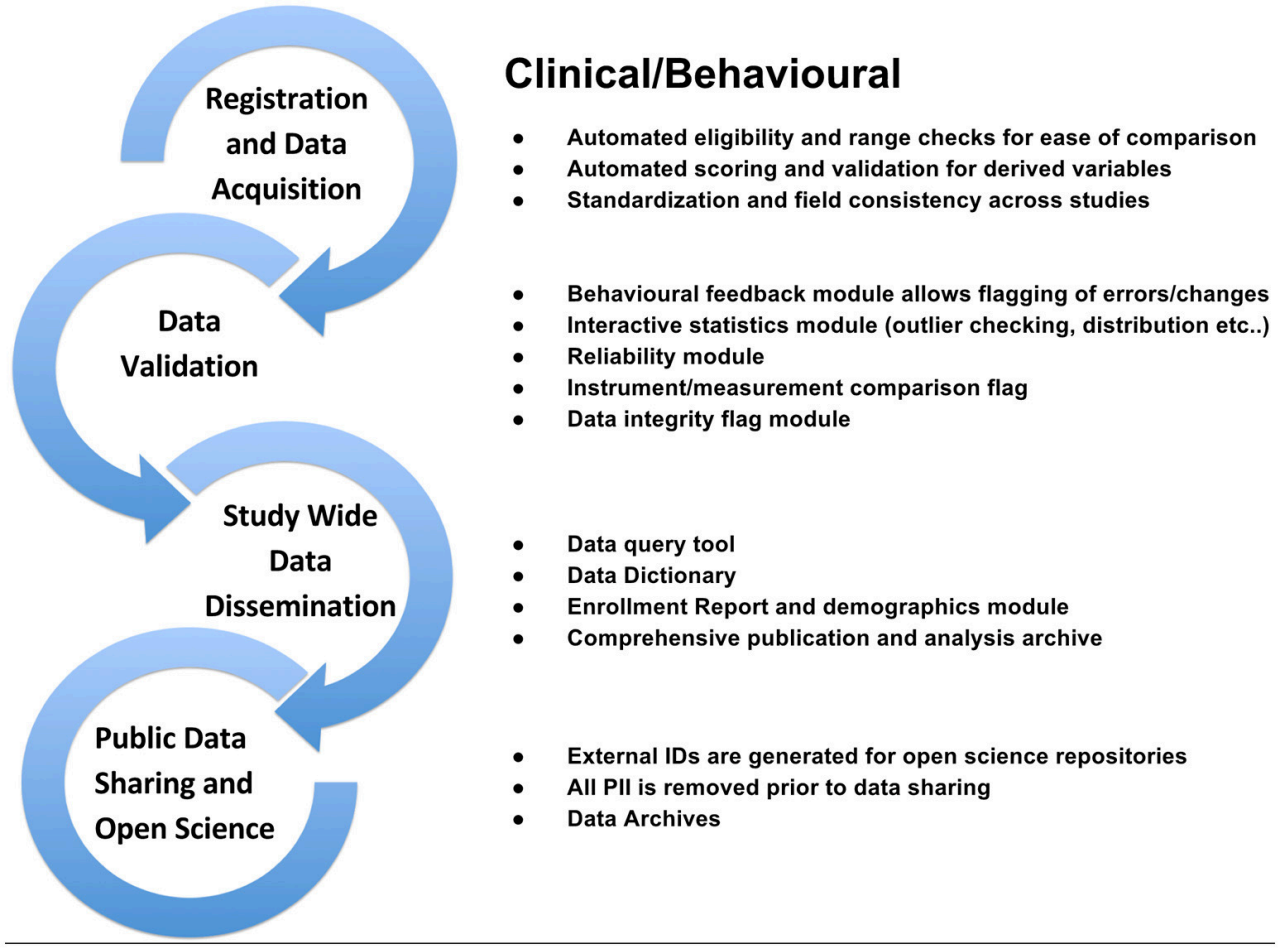
**Clinical/Behavioral workflow from subject registration to data sharing in Open Science detailing data validation, range checks, data integrity flags, and interactive statistics interface at the study and institutional levels**.

To support data review processes, multiple tiers of quality control tools are embedded in LORIS, enabling researchers to standardize data collection, which in turn facilitates reproducible results and compatible data-sharing in an Open Science environment. Validating the reliability of assessments for data collected at different sites and over time enables researchers to control for variability (Van Essen et al., 2013; Ducharme et al., 2015; Orban et al., 2015). Figures 3–5 show domain-specific procedures that allow for data to be both standardized within a study and across studies in the context of Open Science for imaging (Figure 3), biobanking (Figure 4), and clinical/behavioral (Figure 5) data collection.

LORIS implements these new frameworks, techniques, and procedures, both automatic and manual, to ensure that the integrity, validity and reliability of data are not compromised from the collection stage through to data sharing.

### High-performance data processing

Open Science at the MNI is further facilitated by the interface between LORIS and CBRAIN's high performance computing (HPC) capabilities (Das et al., 2016). CBRAIN is a web-based collaborative research platform developed in response to the challenges raised by data-heavy, computationally-intensive neuroimaging research (Sherif et al., 2014). It offers transparent access to remote data sources, distributed computing sites, and an array of processing and visualization tools within a controlled, secure environment. The framework code is entirely open-source and available on GitHub^7^.

**Figure 6:**
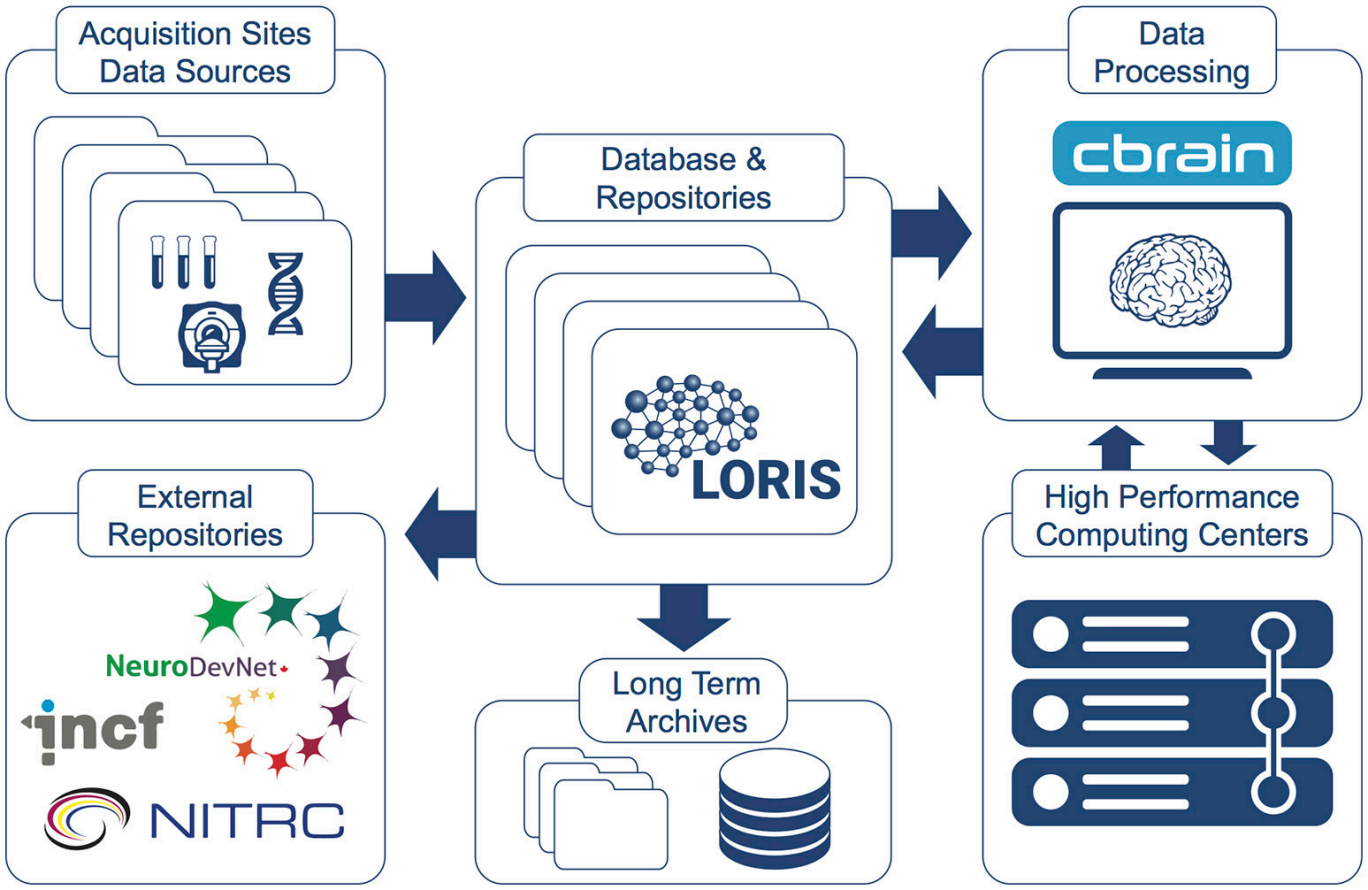
**LORIS and CBRAIN interaction (Das et al., 2016)**. Datasets hosted in LORIS' data-sharing platform are pushed to, processed by, and returned to the central LORIS repository from the CBRAIN distributed computing platform. Data can be downloaded or disseminated at any stage. Custom tools and pipelines can be packaged and mounted on CBRAIN for use by a research group or larger community of investigators.

CBRAIN promotes Open Science in several ways by providing: (1) web access to a wide range of data processing pipelines, (2) an API open to other systems such as LORIS, (3) a full provenance trail of software versions, processing logs and all data manipulations, (4) strong security features, (5) a mechanism of tool containers and descriptors to facilitate the integration and open distribution of new analysis tools/pipelines (Glatard et al., 2015), and (6) connections to new private or shared data sources for research groups. An overview of CBRAIN's integration with LORIS is shown in Figure 6 and further detailed in “The MNI data-sharing and processing ecosystem” (Das et al., 2016).

While LORIS stores and manages the data gathered and distributed by the institute, the CBRAIN platform provides an interface to the tools and high-performance computing and processing capabilities needed by the researchers. CBRAIN and LORIS each have APIs and can be connected such that data files managed by LORIS can be transferred to CBRAIN and processed on its computing ecosystem. When submitted workloads are completed, the resulting data files can be transferred back and registered in LORIS under the proper subject profile. This eliminates the complexity of manual multi-site data transfers and saves the researchers from having to deal with the peculiarities of each computing center (e.g., queueing system and library environment, site policies, number of usable cores per nodes, queue limits, downtime, etc.).

A built-in mechanism allows for extensive provenance recording of any entity managed by CBRAIN, in particular for all operations on files, tasks, user groups, data, and computing resources, as well as the full standard and error logs provided by the analysis tools during processing. This audit trail is essential to ensure future reproducibility of results, and is also useful for troubleshooting and debugging.

CBRAIN's capabilities integrate well with the institutional requirements of privacy when dealing with files that are not yet openly releasable. All CBRAIN data traffic to and from the high performance computing centers is encrypted. Secure connections between authorized resources are transient, and temporary files can be configured to be automatically purged after processing is finished. Fine-grained access rights can be defined on any data file via user groups. Strict access permissions can also be defined for complete data servers, for analysis tools and for computation sites.

Extensibility is an important component of the CBRAIN architecture, and includes software and processing pipelines, data sources and data formats, and computational backends. Researchers can provide different software packages that make a vast number of processing tools available to authorized users. Standardized processing pipelines can be integrated either by writing dedicated CBRAIN plugins, or by leveraging the open Boutiques^8^ framework (Glatard et al., 2015). Boutiques provides a high-level specification to describe command-line tools without writing any code, and to install these tools uniformly on computing systems through Docker^9^ containers. CBRAIN is designed to provide a generic data processing framework, accepting different data-types from various sources as determined by the data-processing software. This is achieved by the creation of data models that associate each data-type with its own processing software and corresponding visualization tool. Finally, CBRAIN provides a meta-scheduler and adaptors to common cluster systems (PBS, Torque, SGE, MOAB, LSF, Amazon EC2 or simple UNIX prompt submission) in order to extend the computational backends needed to process large amounts of data through these diverse processing pipelines.

Currently, CBRAIN deploys Docker containers on a 20,000-node computing cluster provided by Compute Canada, and on Amazon Elastic Compute Cloud (EC2) using its cloud support plugin. Several data analysis tools and processing pipelines are currently deployed in these clusters (CIVET, FreeSurfer, FSL, etc.), and data models for viewing and processing common file types (csv, txt) and various neuroimaging data formats are defined (MINC, NIfTI, BIDS). In the future, other types of containers, for instance Singularity^10^, can further facilitate sharing of new tools in an Open Science context. Other scheduling systems can be easily added using the modularity of the resource access framework to further extend the computational backend.

## Results

The cyberinfrastructure for Open Science at the MNI consists of three primary components: the technical infrastructure that facilitates acquisition, storage, querying, processing, and data analysis; the workflows, procedures and best practices associated with data integrity and privacy at each step; and the data themselves.

### Technical infrastructure

Numerous large-scale projects have already employed LORIS for multi-site use (Evans and Brain development cooperative group, 2006; Wolff et al., 2012; Amunts et al., 2013; Paolozza et al., 2014; Foster et al., 2015; Orban et al., 2015), and several institutions have chosen or planned for LORIS as their institutional infrastructure (e.g., PERFORM Centre at Concordia University, University of Edinburgh's Brain Research Imaging Centre). LORIS is used across 150 acquisition sites in numerous countries with over 500 instruments, over 75,000 variables, and 40 TB of data.

The CBRAIN service deployed at the MNI^11^ currently provides over 460 collaborators in 20 countries with web access to several systems, including six clusters of the Compute Canada^12^ high-performance computing infrastructure (totalling more than 100,000 computing cores and 40PB of disk storage) and Amazon EC2. Presently, CBRAIN transiently stores about 10 million files representing over 50TB distributed over 42 servers. 56 data processing tools are integrated and over 340,000 processing batches have been submitted since 2010.

### Workflows

One of the most important aspects in constructing large-scale data sharing initiatives is the incorporation of properly-designed user workflows, which are vital to ensuring effective usability and viability. Creating software that provides a seamless user experience for a subset of functionalities is a widely understood best practice; however, incorporating diversified workflows into a complex infrastructure, such as institutional Open Science, requires more than wizardry in programming or knowledge of the latest code libraries.

To that end, detailed workflows have been created to facilitate procedures involved in acquiring, storing, and analyzing neuroscience data including clinical, imaging, genetic, and biobanking information. These workflows, outlined in the Methods section of this paper (Figures 3–5), are designed to improve consistency within studies and are critical in an Open Science model across studies. Such procedures help ensure consistency and compliance with data collection standards (i.e., naming, data collection, and imaging pipelines), and coupled with proper and intuitive data organization, provide the foundation of data sharing, for easier interoperability between software systems. Consistent application of such workflows also serves to reduce time spent manually identifying and addressing variability in data formatting. These systems are augmented by a comprehensive set of previously-discussed QC procedures ensuring validation of data and flagging of data for correction. As imaging, clinical, or biospecimen information proceeds from registration through analysis, these streamlined workflows save significant time and energy for researchers as well as developers, all while producing a robustly documented and well-validated dataset.

### The data

Various data types are stored in LORIS including phenotypic, clinical, demographic, imaging, and genomic data. The MNI's Open Science platform will initially consist of contributions of imaging and biobanking data from two key institutional resources. Within the MNI, biospecimens will be housed and tracked in the institutional biobank component of the C-BIG Repository. Neuroimaging data will also be contributed to the C-BIG Repository by researchers using the MNI's McConnell Brain Imaging Centre (BIC) Imagebank platform. The resulting unified repository (see Table 1) will serve the MNI with an enriched data platform, providing multi-modal data querying via the DQT, and enabling visualizations and analyses of more complex datasets (European Society of Radiology, 2015).

**Table 1:** **C-BIG repository overview**.

**MNI C-BIG Centralized LORIS Repository**		
**Type**	**Description**	**Data**
Imagebank	Multi-modal, raw/processed neuroimaging data	MRI, PET, MEG, EEG, Spectroscopy
Biobank	Biospecimen data	Blood, saliva, skin, muscle & nerve biopsies, whole brains, cerebrospinal fluid
Genetic	Summary genetic data	SNPs, CNVs, CpG, GWAS
Phenotypic	Behavioral, clinical data	Instruments, Assessments, Questionnaires

*Data types and description of data that will be stored in the MNI's C-BIG Repository*.

#### Imagebank infrastructure

In its pilot phase, the MNI's Imagebank will serve as a central repository of scans primarily collected at the BIC's MRI unit. Scans transferred to the Imagebank server will be loaded through a series of software scripts into LORIS, and automatically made available for download through the Imagebank's web-based browser interface. This repository allows all images, whether raw or processed, to be available for visualization, quality control, and download/export. Currently, this database links to a compressed archive of every MRI dataset sent to the server, which will grow considerably as the infrastructure is further deployed and usage grows. Expansion for other imaging modalities across the MNI, such as PET and MEG (Niso et al., 2016), is underway. Imaging volumes stored in this LORIS-based repository can be pushed to CBRAIN for image processing and returned in an automated manner into the Imagebank. In addition to storing, processing, archiving, and retrieving data, investigators will have the option of releasing their scans to the Open MNI platform in accordance with institutional ethical and policy constraints as discussed in the Methods section.

#### Biobank infrastructure

Biosamples or biospecimens collected from subjects at the MNI are stored within an infrastructure of freezers and labs. This physical infrastructure, together with the software modules within LORIS which retrieve and process data related to these biospecimens, are collectively referred to as “The Biobank.” Biosample types collected on-site include blood, saliva, skin, muscle, and nerve biopsies, whole brains, and cerebrospinal fluid. LORIS logs specimen information - including sample type, specimen quantity and availability, methodology employed, and so on - beginning at the stage of collection and initial storage and continuing through successive stages of analysis in the research data lifecycle. During these stages, samples may also be located offsite in any number of collaborating institutions or facilities, such as the Genome Quebec Innovation Centre. Results from the assays and analysis performed on these specimens are stored in LORIS.

Both qualitative and quantitative outputs - such as cell counts, protein expression, or diagnostic information—can be captured for each biospecimen. Precisely which input fields are used depends on the study and can be extended and customized on a per-project and/or per-methodology basis. All of these data are queryable in conjunction with clinical/behavioral data which are also stored in LORIS.

LORIS contains a wide range of data collected from physical biospecimens, including skin, blood and saliva. In addition to these common sample types, a key strength of the MNI biobank is enabling access to data obtained via complex, invasive or rare procedures, such as muscle, brain and nerve biopsies, cerebrospinal fluid, and whole brain specimens. Information and analyses collected by one researcher (including data acquisition log files, observations, models, outcomes, etc.) can be added to the biobank for review and reuse by others. In providing access to a large online dataset, LORIS greatly facilitates optimal use and data re-analysis of rare specimens. This has clear benefits for the acceleration of new discoveries in neuroscience.

## Discussion

Open Science, at an institutional level, is a concept that has not yet been widely adopted across the scientific community. In tandem with the deployment of a robust cyberinfrastructure, key enhancements to organizational practices are necessary for Open Science to truly proliferate. Beginning with obtaining subject consent for data sharing, protecting subject privacy and complying with ethical regulations, there are challenges in ensuring that all such considerations are executed properly, securely, and effectively.

For an institution to go completely open, it requires considerable buy-in from investigators who will share data and tools, and a comprehensive institutional policy contingent upon full support and leadership across the organization. Naturally there are some risks and challenges associated in the adoption of an Open Science framework. On an individual level, researchers may be concerned about the ownership of data they have generated, or autonomy over their research findings. However the realities of any such risks are far outweighed by increasing the outreach of the research and the number of citations (Piwowar and Vision, 2013) and recognition that is attributed to shared data, as initiatives such as ADNI (Petersen et al., 2010), the Human Connectome Project (Van Essen et al., 2012), ABIDE (Di Martino et al., 2014), FCP (Biswal et al., 2010), ADHD (ADHD-200 Consortium, 2012), OpenfMRI (Poldrack et al., 2013), and CoRR (Zuo et al., 2014) have demonstrated. From an institutional perspective, there is often a fear that foregoing potential patent royalties will result in lost revenue and recognition of innovation (David, 2004). However, open access initiatives can result in greater funding opportunities, increased efficiency, and greater institutional recognition (Poldrack and Gorgolewski, 2014).

The MNI's commitment to move toward an Open Science model of data sharing (Owens, 2016a,b) leverages the benefits of increased access to datasets in sample sizes and variability while advancing the data lifecycle toward enriching exploratory analyses and hypothesis formulation, which allows for new questions to be asked. Increased sample size and sample variation also improves reproducibility and reliability of inference testing as well as publication quality and impact. While simply releasing data under an Open Science context does not in itself address all the concerns regarding reproducibility (such as selective reporting and analysis, processing pipeline deviations, proper documentation, etc.), it does push toward principles of replicability by pressuring for improved descriptions and provenance, allowing for increased analysis and re-analysis, and facilitating collaborative quality control and validation (Zuo et al., 2014; Zuo and Xing, 2014).

It is important to note that by facilitating collaborations through data sharing, the cost of entry for many researchers will be lowered (Edwards et al., 2009; Abboud, 2016; Owens, 2016a,b), thus maximizing the return on public science funding and research investments (Poldrack and Gorgolewski, 2014). Emerging interoperability between specialized data systems, such as XNAT (imaging, Marcus et al., 2007), REDCap (clinical/behavioral, Harris et al., 2009) and LIMS systems, as well as LORIS, will also serve to lower technical barriers to the federation of datasets across modalities and repositories.

Another important consideration for Open Science at the MNI is its foundation on an established software infrastructure—i.e., the combination of LORIS and CBRAIN—that has been already operational for several years. Over the lifecycle of these applications, these platforms have been designed and developed in close collaboration with researchers and have grown according to their needs and goals. This infrastructure is used internationally, operating across the full life-cycle of data-sharing (i.e., acquisition to analysis), and is proven to be scalable for large-scale datasets. This wealth of experience is key to the cyberinfrastructure of the Open Science initiative as it addresses many of the major hurdles that this endeavor could involve. However, as the first of its kind, the MNI's institutional Open Science initiative has necessitated the addition of the following features and functionalities.

In LORIS:

1. A complete de-identification mechanism has been developed that allows publication of data beyond the usual confines of a particular study, while at the same time ensuring ethics and privacy.
2. Support for several data modalities is being added, including PET, EEG/MEG, and biosamples. This is of particular importance since the range of modalities used at an institutional level is much wider than in a single project.
3. Quality control tools have been extended and made more robust, based on 15 years of experience in a number of data acquisition project lifecycles.

In CBRAIN:

1. Tighter integration with the LORIS database to allow for compute-intensive processing of Open Data.
2. Streamlined account creation process and handling of access permissions, so that various user profiles can be easily handled by administrators. This will be particularly important when the MNI's Open Science initiative reaches its full potential, as users with a wide range of profiles are expected to access the data and to have various processing requirements.
3. Facilitated tool integration, so that external researchers could contribute their tool to the CBRAIN ecosystem without expert knowledge of its internal mechanisms.

## Conclusion

Open Science is a simple concept that masks a daunting set of ethical, conceptual, and technical challenges. As the scale of scientific data collection and scope of discovery increase with technological advancement, the promise of collaboration through Open Science presents a potential solution to limits faced by institution-based science, including statistical power and resource constraints. This Open Science cyberinfrastructure at the MNI, comprised of the LORIS and CBRAIN platforms, intends to increase transparency in data curation, dissemination and analysis, reduce data loss, facilitate innovation and collaboration, and efficiently accelerate the discovery and the application of neuroscience at the Montreal Neurological Institute and across the greater research community.

## Author contributions

SD, TG, MR, AE—Contributed to the writing of this paper, contributed to the infrastructure, contributed to conceptualization of the initiative, contributed to policy. JK, AB, RH, EF, GR—Contributed to the writing of this paper, contributed to conceptualization of the initiative, contributed to policy. CR, JSA, SP, DM, JST, PR, SM, PK—Contributed to the writing of this paper, contributed to the infrastructure, contributed to conceptualization of the initiative. LM, MS, VF, IL, TC—Contributed to the writing of this paper, contributed to the infrastructure. CM, ZM, XL, DC—Contributed to the infrastructure, contributed to conceptualization of the Initiative. AD, DC, SB—Contributed to the writing of this paper, contributed to conceptualization of the initiative. CD, SV—contributed to conceptualization of the initiative, contributed to policy. RA, NM, TD, JM—contributed to conceptualization of the initiative.

### Conflict of interest statement

The authors declare that the research was conducted in the absence of any commercial or financial relationships that could be construed as a potential conflict of interest. The reviewer BG and handling Editor declared their shared affiliation, and the handling Editor states that the process nevertheless met the standards of a fair and objective review.

## Footnotes

^1^Open Science (Open Access). HORIZON 2020, The EU Framework Programme for Research and Innovation. Retrieved from https://ec.europa.eu (Accessed on August 29, 2016).

^2^PBKDF2 is a key derivation function that applies a pseudo-random function to a specified input, repeating the process multiple times, to produce a derived key (https://en.wikipedia.org/wiki/PBKDF2).

^3^SHA-1 a cryptographic hash function designed as a one-way function to map data of arbitrary size to a fixed data size, making it unfeasible to invert. It is considered a U.S. Federal Information Processing Standard (https://en.wikipedia.org/wiki/SHA-1).

^4^https://github.com/aces/Loris

^5^https://github.com/aces/Loris-MRI

^6^https://github.com/aces/brainbrowser

^7^https://github.com/aces/cbrain

^8^http://boutiques.github.io

^9^https://docker.com

^10^http://singularity.lbl.gov

^11^https://portal.cbrain.mcgill.ca

^12^http://www.computecanada.ca
